# Supplementary material for: Heat adaptation of phage T7 under an extended genetic code
Source: Virus Evol. 2021 Dec 1;7(2):veab100. doi: 10.1093/ve/veab100 (PMC8923235; doi:10.1093/ve/veab100)
Supplement: veab100_Supp [file veab100_supp.zip › Sequencing_Analysis.zip!/Sequencing_Analysis/S3/evidence/SNP_12.html]

BRESEQ :: Evidence


| Predicted mutation | | | | | | |
| --- | --- | --- | --- | --- | --- | --- |
| evidence | seq id | position | mutation | annotation | gene | description |
| RA | NC\_001604.1 | 29,258 | A→G | intergenic (–/–) | – / – | –/– |

| Read alignment evidence... | | | | | | | | | | | |
| --- | --- | --- | --- | --- | --- | --- | --- | --- | --- | --- | --- |
|  | seq id | position | | ref | new | freq | score (cons/poly) | reads | annotation | genes | product |
| \* | NC\_001604.1 | 29,258 | 0 | A | G | 99.7% | 4662.3 / NA | 1532 | intergenic (–/–) | *–/–* | –/– |
| Reads supporting (aligned to +/- strand):  **ref** base A (0/3);  **major** base G (801/722);  **minor** base T (3/1);  **total** (805/727) | | | | | | | | | | | |
| Fisher's exact test for biased strand distribution *p*-value = 6.27e-01 | | | | | | | | | | | |
| Kolmogorov-Smirnov test that lower quality scores support variant *p*-value = 1.00e+00 | | | | | | | | | | | |

Only 100 of 1535 total aligned reads displayed.  

`GCGTCGTCCAGACTCTGCTGACTTCTTTGAGAAGTATATCGACAACGGTCTGGTTACTGGCGCAATCCCATCTGATGCTCAAGCCACACAGCTTATAAGCCAAGCGTTCAGTGACGCTTCTAGCCGTGCTGGTGGTGCTGACTTCCTGATGCGAGTCGGTGACAAGAAGGTAACACTTAACGGAGCCACTACGACTTACCGAGAGTTGATTGGTGAGGAACAGTGGAACGCTCTCATGGTCACAGCACAACGTTCTCAGTTTGAGACTGACGCGAAGCTGAACGAGCAGTATCGCTTGAAGATTAACTCTGCGCTGAACCAAGAGGACCCAAGGACAGCTTGGGAGATGCTTCAAGGTATCAAGGCTGAACTAGATAAGGTCCAACCTGATGAGCAGATGACACCACAACGTGAGTGGCTAATCTCCGCACAGGAACAAGTTCAGAATCAGATGAACGCATGGACGAAAGCTCAGGCCAAGGCTCTGGACGATTCCATGAAGTCAATGAACAAACTTGACGTAATCGACAAGCAATTCCAGAAGCGAATCAACGGTGAGTGGGTCTCAACGGATTTTAAGGATATGCCAGTCA  >  NC_001604.1/28960‑29552`  

                                                                                                                                                                                                                                                                                                          `|`                                                                                                                                                                                                                                                                                                        
`gcGTCGTCCAGACTCTGCTGACTTCTTTGAGAAGTATATCGACAACGGTCTGGTTACTGGCGCAATCCCATCTGATGCTCAAGCCACACAGCTTATAAGCCAAGCGTTCAGTGACGCTTCTAGCCGTGCTGGTGGTGCTGACTTCCTGATGCGAGTCGGTGACAAGAAGGTAACACTTAACGGAGCCACTACGACTTACCGAGAGTTGATTGGTGAGGAACAGTGGAACGCTCTCATGGTCACAGCACAACGTTCTCAGTTTGAGACTGACGCGAAGCTGAACGAGCAGTATCGCTTGGAg                                                                                                                                                                                                                                                                                                      <  1:285131/301‑1 (MQ=255)`  
     `gtcCAGACTCTGCTGACTTCTTTGAGAAGTATATCGACAACGGTCTGGTTACTGGCGCAATCCCATCTGATGCTCAAGCCACACAGCTTATAAGCCAAGCGTTCAGTGACGCTTCTAGCCGTGCTGGGGGTGCTGACTTCCTGATGCGAGTCGGTGACAAGAAGGTAACACTTAACGGAGCCACTACGACTTACCGAGAGTTGATTGGTGATGGACAGTGGAACGCTCTCATGGTCACAGCACAACGTTCTCAGTTTGAGACTGACGAGCAGATGAACGCGCAGTCACGCTTGGGGATTaa                                                                                                                                                                                                                                                                                                 >  1:64237/1‑301 (MQ=255)`  
      `tcCAGACTCTGCTGACTTCTTTGAGAAGTATATCGACAACGGTCTGGTTACTGGCGCAATCCCATCTGATGCTCAAGCCACACAGCTTATAAGCCAAGCGTTCAGTGACGCTTCTAGCCGTGCTGGTGGTGCTGACTTCCTGATGCGAGTCGGTGACAAGAAGGTAACACTTAACGGAGCCACTACGACTTACCGAGAGTTGATTGGTGAGGAACAGTGGAACGCTCTCATGGTCACAGCACAACGTTCTCAGTTTGAGACTGACGCGAAGCTGAACGAGCAGTATCGCTTGGAGATTAAc                                                                                                                                                                                                                                                                                                <  1:266421/301‑1 (MQ=255)`  
        `cAGACTCTGCTGACTTCTTTGAGAAGCATATCGACAACGGTCTGGTTACTGGCGCAATCCCATCTGATGCTCAAGCCACACAGCTTATAAGCCAAGCGTTCAGTGACGCTTCTAGCCGTGCTGGTGGTGCTGACTTCCTGATGCGAGTCGGTGACAAGAAGGTAACACTTAACGGAGCCACTACGACTTACCGAGAGTTGATTGGTGAGGAACAGTGGAACGCTCTCATGGTCACAGCACAACGTTCTCAGTTTGAGACTGACGCGAAGCTGAACGAGCAGTATCGCTTGGAGATTAActc                                                                                                                                                                                                                                                                                              <  1:144217/301‑1 (MQ=255)`  
           `aCTCTGCTGACTTCTTTGAGAAGTATATCGACAACGGTCTGGTTACTGGCGCAATCCCATCTGATGCTCAAGCCACACAGCTTATAAGCCAAGCGTTCAGTGACGCTTCTAGCCGTGCTGGTGGTGCTGACTTCCTGATGCGAGTCGGTGACAAGAAGGTAACACTTAACGGAGCCACTACGACTTACCGAGAGTTGATTGGTGAGGAACAGTGGAATGCTCTCATGGTCACAGCACAACGTTCTCAGTTGGAGACTGAGGCGAAGCTGAACGAGCAGAAACGCTTGGAGATTAACTCTgc                                                                                                                                                                                                                                                                                           >  1:274209/1‑301 (MQ=255)`  
             `ctggcttACTTCTTTGAAAATTATATCGACACCGGTCTGGTTACTGGCGCATTCCCATCTGATGCTCAAGCCACACAGCTTATAAGCCAAGCGTTCAGTGACGCTTCTAGCCGTGCTGGTGGTGCTGACTTCCTGATGCGAGTCGGTTACAAGAAGGTAACACTTAACGGAGCCACTACGACTTACCGAGAGTTGATTGGTGAGGAACAGTGGAACGCTCTCATGGTCACAGCACAACGTTCTCAGTTTGAGACTGACGCGAAGCTGAACGAGCAGTATCGCTTGGAGATTAACTCTgcgc                                                                                                                                                                                                                                                                                         <  1:220007/298‑1 (MQ=255)`  
              `ctgctgACTTCTTTGAGAAGTATATCGACAACGGTCTGGTTACTGGCGCAATCCCATCTGATGCTCAAGCCACACAGCTTATAAGCCAAGCGTTCAGTGACGCTTCTAGCCGTGCTGGTGGTGCTGACTTCCTGATGCGAGTCGGTGACAAGAAGGTAACACTTAACGGAGCCACTACGACTTACCGAGAGTTGATTGGTGAGGAACAGTGGAACGCTCTCATGGTCACAGCACAACGTTCTCAGTTTGAGACTGACGCGAAGCTGAACGAGCAGTATCGCTTGGAGATTAACTCTGCGCt                                                                                                                                                                                                                                                                                        >  1:200998/1‑301 (MQ=255)`  
                   `gACTTCTTTGAGAAGTATATCGACAACGGTCTGGTTACTGGCGCAATCCCATCTGATGCTCAAGCCACACAGCTTATAAGCCAAGCGTTCAGTGACGCTTCTAGCCGTGCTGGTGGTGCTGACTTCCTGATGCGAGTCGGTGACAAGAAGGTAACACTTAACGGAGCCACTACGACTTACCGAGAGCTGATTGGTGAGGAACAGTGGAACGCTCTCATGGTCACAGCACAACGTTCTCAGTTTGAGACTGACGCGAAGCTGAACGAGCAGTATCGCTTGGAGATTAACTCTGCGCTGAAcc                                                                                                                                                                                                                                                                                   <  1:287952/301‑1 (MQ=255)`  
                    `aCTTCTTTAAGAAGTATATCGACAACGGTCTGGTTACTGGCGCAATCCCATCTGATGCTCAAGCCACACAGCTTATAAGCCAAGCGTTCAGTGACGCTTCTAGCCGTGCTGGTGGTGCTGACTTCCTGACGCGAGTCGGTGACAAGAAGGTAACACTTAACGGAGCCACTACGACTTACCGAGAGTTGATTGGTGAGAAACAGTGGAACGCTCTCATGGTCACAGCACAACGTTCTCAGTTTGAGACTGACGCGAAGCTGAACGAGCAGTATCGCTTGGAGATTAACTCTGCGCTGAACCa                                                                                                                                                                                                                                                                                  <  1:100738/301‑1 (MQ=255)`  
                       `tcttTGAGAAGTATATCGACAACGGTCTGGTTACTGGCGCAATCCCATCTGTTGCTCAAGCCCCCCAGCTTATAAGCCAAGCGTTCATTGACGCTTCTAGCCGTGCTGGTGGTGCTGACTTCCTGTTGCGAGTCGGTGACAAGACGGTAACACTTAACTGAGCCACTACGACTTACCGATAGTTGATTGGTGTGGAACAGTGGAACGCACTCATGGTCACAGCACCACGGTCTCAGTTTGGGACTGACGCGAAGCTGAACGAGCAGTATCGCTTGGAGATTAACTCTGCGCTGAACCAagg                                                                                                                                                                                                                                                                               >  1:324177/1‑300 (MQ=255)`  
                       `ataagagacaGTATATCGACAACGGTCTGGTTACTGGCGCAATCCCATCTGATGCTCAAGCCACACAGATTATAAGCCAAGCGTTCAGTGACGCTTCTAGCCGTGCTGGTGGTGCTGACTTCCTGATGCGAGTCGGTGACAAGAAGGTAGCACTTAACGGAGCCACTACGACTTACCGAGAGTTGATTGGTGAGGAACAGTGGAACGCTCTAATGGTCACAGCACAACGTTCTCAGTTTGAGACTGACGCGAAGCTGAACGAGCAGTATCGCTTGGAGATTAACTCTGCGCTGAACCAaga                                                                                                                                                                                                                                                                               <  1:59604/292‑1 (MQ=255)`  
                              `gaAGTATATCGACAACGGTCTGGTTACTGGCGCAATCCCATCTGATGCTCAAGCCACACAGCTTATAAGCCAAGCGTTCAGTGACGCTTCTAGCCGTGCTGGTGGTGCTGACTTCCTGATGCGAGTCGGTGACAAGAAGGTAACACTTAACGGAGCCACTACGACTTACCGAGAGTTGATTGGTGAGGAACAGTGGAACGCTCTCATGGTCACAGCACAACGTTCTCAGTTTGAGACTGACGCGAAGCTGAACGAGCAGTATCGCTTGGAGATTAACTCTGCGCTGAACCAagactgtctc                                                                                                                                                                                                                                                                        >  1:299387/1‑294 (MQ=255)`  
                              `gaAGTATATCGACAACGGTCTGGTTACTGGCGCAATCCCATCTGATGCTCAAGCCACACAGCTTATAAGCCAAGCGTTCAGTGACGCTTCTAGCCGTGCTGGTGGTGCTGACTTCCTGATGCGAGTCGGTGACAAGAAGGTAACACTTAACGGAGCCACTACGACTTACCGAGAGGTGATTGGTGAGGAACAGTGGAACGCGCTCATGGTCACAGCACAACGTTCTCAGTTTGAGACTGACGCGAAGCTGAACGAGCAGTATCGCTTGGAGATTAACGCTGCGCTGAACCAAGAGGAccaa                                                                                                                                                                                                                                                                        >  1:93507/1‑299 (MQ=255)`  
                                `aGTATAGCGACAACGGTCTGGTTACTGGCGCAATCCCATCTGATGCTCAAGCCACACAGCTTATAAGCCAAGCGTTCAGTGACGCTTCTAGCCGTGCTGGTGGTGCTGACTTCCTGATGCGAGTCGGTGACAAGAAGGTAACACTTAACGGAGCCACTACGACTTACCGAGAGTTGATTGGTGAGGAACAGTGGAACGCTCTCATGGTCACAGCACAACGTTCTCAGTTTGAGACTGACGCGAAGCTGAACGAGCAGTATCGCTTGGAGATTAACTCTGCGCTGAACCAAGAGGACCCAAg                                                                                                                                                                                                                                                                      <  1:4680/301‑1 (MQ=255)`  
                                 `gTATATCGACAACGGTCTGGTTACTGGCGCAATCCCATCTGATGCTCAAGCCACACAGCTTATAATCCAAGCGTTCAGTGACGCTTCTAGCCGTGCTGGTGGTGCTGACTTCCTGATGCGAGTCGGTGACAAGAAGGTAACACTTAACGGAGCCACTACGACTTACCGAGAGTTGATTGGTGAGGAACAGTGGAACGCTCTCATGGTCACAGCACAACGTTCTCAGTTTGAGACTGACGCGAAGCTGAACGAGCAGTATCGCTTGGAGATTAACTCTGCGCTGAACCAAGAGGACCCAAgg                                                                                                                                                                                                                                                                     >  1:280594/1‑301 (MQ=255)`  
                                 `gTATATCGACAACGGTCTGGTTACTGGCGCAATCCCATCTGATGCTCAAGCCACACAGCTTATAAGCCAAGCGTTCAGTGACGCTTCTAGCCGTGCTGGTGGTGCTGACTTCCTGATGCGAGTCGGTGACAAGAAGGTAACACTTAACGGAGCCACTACGACTTACCGAGAGTTGATTGGTGAGGAACAGTGGAACGCTCTCATGGTCACAGCACAACGTTCTCAGTTTGAGACTGACGCGAAGCTGAACGAGCAGTATCGCTTGGAGATTAACTCTGCGCTGAACCAAGAGGACCCAAgg                                                                                                                                                                                                                                                                     >  1:226288/1‑301 (MQ=255)`  
                                  `tataTCGACAACGGTCTGGTTACTGGCGCAATCCCATCTGATGCTCAAGCCACACAGCTTATAAGCCAAGCGTTCAGTGACGCTTCTAGCCGTGCTGGTGGTGCTGACTTCCTGATGCGAGTCGGTGACAAGAAGGTAACACTTAACGGAGCCACTACGACTTACCGAGAGTTGATTGGTGAGGAACAGTGGAACGCTCTCATGGTCACAGCACAACGTTCTCAGTTTGAGACTGACGCGAAGCTGAACGAGCAGTATCGCTTGGAGATTAACTCTGCGCTGAACCAAGAGGACCCAAGGa                                                                                                                                                                                                                                                                    >  1:334848/1‑301 (MQ=255)`  
                                   `ataagagacagggTGTGGTTACTGGCGCAAGCCCGTCTGATGCTCAAGCCACACAGCTTATAAGCCAAGCGTTCAGTGACGCTTCTAGCCGTGCTGGTGGTGCTGACTTCCTGATGCGAGTAGGTGACAAGAAGGTAACACTTAACGGAGCCACTACGACTTACCGAGAGTTGATTGGTGAGGAACAGTGGAACGCTCTCATGGTCACAGCACAACGTTCTCAGTTTGAGACTGACGCGAAGCTGAACGAGCAGTATCGCTTGGAGATTAACTCTGCGCTGAACCAAGAGGACCCAAGGAc                                                                                                                                                                                                                                                                   <  1:198803/290‑1 (MQ=255)`  
                                   `agacagaaaaCGGTCTGGTTACTGGCGCAATCCCATCTGATGCTCAAGCCACACAGCTTATAAGCCAAGCGTTCAGTGACGCTTCTAGCCGTGCGGGTGGTGCTGACTTCCTGATGCGAGTCGGTGACAAGAAGGTAACACATAACGGAGCCACTACGACTTACCGAGAGTTGATTGGTGAGGAACAGTGGAACGCTCTCATGGTCACAGCACAACGTTCTCAGTTTGAGACTGACGCGAAGCTGAACGAGCAGTATCGCTTGGAGATTAACTCTGCGCTGAACCAAGAGGACCCAAGGAc                                                                                                                                                                                                                                                                   <  1:315071/293‑1 (MQ=255)`  
                                            `aCGGTCTGGTTACTGGCGCAATCCCATCTGATGCTCAAGCCACACAGCTTATAAGCCAAGCGTTCAGTGACGCTTCTAGCCGTGCTGGTGGTGCTGACTTCCTGATGCGAGTCGGTGACAAGAAGGTAACACTTAACGGAGCCACTACGACTTACCGAGAGTTGATTGGTGAGGAACAGTGGAACGCTCTCATGGTCACAGCACAACGTTCTCAGTTTGAGACTGACGCGAAGCTGAACGAGCAGTATCGCTTGGAGATTAACTCTGCGCTGAACCAAGAGGACCCAAGGACAGCTTGGga                                                                                                                                                                                                                                                          <  1:303205/301‑1 (MQ=255)`  
                                             `cGGTCTGGTTACTGGCGCAATCCCATCTGATGCTCAAGCCACACAGCTTATAAGCCAAGCGTTCAGTGACGCGTCTAGCCGTGCTGGTGGTGCTGACTTCCTGATGCGAGTCGGTGACAAGAAGGTAACACTTAACGGAGCCACTACGACTTACCGAGAGTTGATTGGTGAGGAACAGTGGAACGCTCTCATGGTCACAGCACAACGTTCTCAGTTTGAGACTGACGCGAAGCTGAACGAGCAGTATCGCTTGGAGATTAACTCTGCGCTGAACCAAGAGGACCCAAGGACAGCTTGGgag                                                                                                                                                                                                                                                         <  1:195496/301‑1 (MQ=255)`  
                                             `cGGTCTGGTTACTGGCGCAATCCCATATGAGGCTCAAGCCACACAGCTTATAAGCCAAGCGTTCAGTGACGCTTCTAGCCGTGCTGGTGGTGCTGACTTCCTGATGCGAGTCGGTGACAAGAAGGTAACACTTAACGGAGCCACTACGACTTACCGAGAGTTGATTGGTGAGGAACAGTGGAACGCTCTCATGGTCACAGCACAACGTTCTCAGTTTGAGACTGACGCGAAGCTGAACGAGCAGTATCGCTTGGAGATTAACTCTGCGCTGAACCAAGAGGACCCAAGGACAGCTTGGgag                                                                                                                                                                                                                                                         <  1:193195/301‑1 (MQ=255)`  
                                               `gTCTGGTTACTGGCGCAATCCCATCTGATGCTCAAGCCACACAGCTTATAAGCCAAGCGTTCAGTGACGCTTCTTCCCGTGCTGGTGGTTCTGACTTCCTTTTTCGAGTCGGTTACCTTTAGGTAACTCTTACCGGAGCCACTCCTACTTACCGAGTGTTCTTTGGTGAGGATCACTGTAACGCTCTCATGGTCACAGCACAACGTTCTCAGTTTGATACTTACGCTAACCTGAACCAGCAGTATCGCTTTGAGTTTAACTCTTCCCTCTACCaatacgctgtctcttatccacatctcct                                                                                                                                                                                                                                                       >  1:83478/1‑275 (MQ=255)`  
                                               `gTCTGGTTACTGGCGCAATCCCATCTGATGCTCAAGCCACACAGCTTATAAGCCAAGCGTTCAGTGACGCTTCTAGCCGTGCTGGTGGTGCTGACTTCCTGATGCGAGTCGGTGACAAGAAGGTAACACTTAACGGAGCCACTACGACTTACCGAGAGTTGATTGGTGAGGAACAGTGGAACGCTCTCATGGTCACAGCACAACGTTCTCAGTTTGAGACTGACGCGAAGCTGAACGAGCAGTATCGCTTGGAGATTAACTCTGCGCTGAACCAAGAGGACCCAAGGACAGCTTGGGAGAt                                                                                                                                                                                                                                                       >  1:112998/1‑301 (MQ=255)`  
                                                        `cTGGCGCAATCCCATCTGATGCTCAAGCCACACAGCTTATAAGCCAAGCGTTCAGTGACGCTTCTAGCCGTGCTGGTGGTGCTGACTTCCTGATGCGAGTCGGTGACAAGAAGGTAACACTTAACGGAGCCACTACGACTTACCGAGAGTTGATTGGTGAGGAACAGTGGAACGCTCTCATGGTCACAGCACAACGTTCTCAGTTTGAGACTGACGCGAAGCTGAACGAGCAGTATCGCTTGGAGATTAACTCTGCGCTGAACCAAGAGGACCCAAGGACAGCTTGGGAGATGCTTCAAgg                                                                                                                                                                                                                                              <  1:141235/301‑1 (MQ=255)`  
                                                              `agaTCCCATCTGATGCTCAAGCCACACAGCTTATAAGCCAAGCGTTCAGTGACGCTTCTAGCCGTGCTGGTGGTGCTGACTTCCTGATGCGAGTCGGTGACAAGAAGGTAACACTTAACGGAGCCACTACGACTTACCGAGAGTTGATTGGTGAGGAACAGTGGAACGCTCTCATGGTCACAGCACAACGTTCTCAGTTTGAGACTGACGCGAAGCTGAACGAGCAGTATCGCTTGGAGATTAACTCTGCGCTGAACCAAGGGGACCCAAGGACAGCTTGGGAGATGCTTCAAGGTATCaa                                                                                                                                                                                                                                        <  1:163916/299‑1 (MQ=255)`  
                                                                `aTCCCATCTGATGCTCAAGCCACACAGCTTATAAGCCAAGCGTTCAGTGACGCTTCTAGCCGTGCTGGTGGTGCTGACTTCCTGATGCGAGTCGGTGACAAGAAGGTAACACTTAACGGAGCCACTACGACTTACCGAGAGTTGATTGGTGAGGAACAGTGGAACGCTCCCATGGTCACAGCACAACGTTCTCAGTTTGAGACTGAAGCGAAGCTGAAAGAGCAGTAACGCTTGGAGATTAACTCTGCGCTGAAACCAGAAGACCCAAGGACAGCATGGGAGATGCCTCCAGGTATCAAgg                                                                                                                                                                                                                                      >  1:186805/1‑301 (MQ=255)`  
                                                                  `cccATCTGATGCTCAAGCCACACAGCTTATAAGCCAAGCGTTCAGTGACGCTTCTAGCCGTGCTGGTGGTGCTGACTTCCTGATGCGAGTCGGTGACAAGAAGGTAACACTTAACGGAGCCACTACGACTTACCGAGAGTTGATTGGTGAGGAACAGTGGAACGCTCTCATGGTCACAGCACAACGTTCTCAGTTTGAGACTGACGCGAAGCTGAACGAGCAGTATCGCTTGGAGATTAACTCTGCGCTGAACCAAGAGGACCCAAGGACAGCTTGGGCGATGCTTCAAGGTATCAAGGCt                                                                                                                                                                                                                                    >  1:174104/1‑301 (MQ=255)`  
                                                                    `cATCTGATGCTCAAGCCACACAGCTTATAAGCCAATCGTTCAGTGACGCTTCTAGCCGTGCTGGTGGTGCTGACTTCCTGATGCGAGTCGGTGACAAGAAGGTAACACTTAACGGAGCCACTACGACTTACCGAGAGTTGATTGGTGAGGAACAGTGGAACGCTCTCATGGTCACAGCACAACGTTCTCAGTTTGAGACTGACGCGAAGCTGAACGAGCAGTATCGCTTGGAGATTAACTCTGCGCTGAACCAAAAGGACCCAAGGACAGCTTGGGAGATGCTTCAAGGTATCAAGGCTGa                                                                                                                                                                                                                                  >  1:130584/1‑301 (MQ=255)`  
                                                                       `cTGATGCTCACGCCACACAGCCTACAAGCCAAGCGTTCAGTGACGCTTCTAGCCGTTCTGGTGGTGCTGACTTCCTGATGCGAGTCGGTGACAAGAAGGTAACACTTAACGGAGCCACTACGACTTACCGAGAGTTGATTGGTGAGGAACAGTGGAACGCTCTCATGGTCACAGCACAACGTTCTCAGTTTGAGACTGACGCGAAGCTGAACGAGCAGTATCGCTTGGAGATTAACTCTGCGCTGAACCAAGAGGACCCAAGGACAGCTTGGGAGATGCTTCAAGGTATCAAGGCTGAACt                                                                                                                                                                                                                               <  1:2424/301‑1 (MQ=255)`  
                                                                           `tgatCAAGCCACACAGCTTATAAGCCAAGCGTTCAGTGACGCTTCTAGCCGTGCTGGTGGTGCTGACTTCCTGATGCGAGTCGGTGACAAGAAGGTAACACTTAACGGAGCCACTACGACTTACCGAGAGTTGATTGGTGAGGAACAGTGGAACGCTCTCATGGTCACAGCACAACGTTCTCAGTTTGAGACTGACGCGAAGCTGAACGAGCAGTATCGCTTGGAGATTAACTCTGCGCTGAACCAAGAGGACCCAAGGACAGCTTGGGAGATGCTTCAAGGTATCAAGGCTGAACTAGAt                                                                                                                                                                                                                           <  1:222638/298‑1 (MQ=255)`  
                                                                           `tGCTCAAGCCACACAGCTTATAAGCCAAGCGTTCAGTGACGCTTCTAGCCGTGCTGGTGGTGCTGACTTCCTGATGCGAGTCGGTGACAAGAAGGTAACACTTAACGGAGCCACTACGACTTACCGAGAGTTGATTGGTGAGGAACAGTGGAACGCTCTCATGGTCACAGCACAACGTTCTCAGTTTGAGACTGACGCGAAGCTGAACGAGCAGTATCGCTTGGAGATTAACTCTGCGCTGAACCAAGAGGACCCAAGGACAGCTTGGGAGATGCTTCAAGGTATCAAGGCTGAACTAGAt                                                                                                                                                                                                                           <  1:90278/301‑1 (MQ=255)`  
                                                                            `gcgaaaGCCACACAGCTTATAAGCCTAGCGTTGAGTGACGCGTCTAGCCGTGCTGGTGGTGCTGACTTGCTGAGGCGAGTCGGTGACAAGAAGGTAAGACTTAACGGAGCCACTAGGAATTACCGAGAGGTGATTGGTGAGGAACAGTGGAACGGTCTCATGGTCACAGCACAGCGTTCTCAGTTTGAGACTGACGAGAAGCTGAACGAGCAGTATCGCTTGGAGATGAACTCTGCGCTGAACCAAGAGGACCCAAGGACAGCTTGGGAGATGCGTCAAGGTATCAAGGCTGAACTAGATa                                                                                                                                                                                                                          <  1:297302/297‑1 (MQ=255)`  
                                                                            `gaacAAGCCACACAGCTTATACGCCAAGCGTTCAGTGACGCTGCTAGACGTGCTGGTGGTGCTGACTTCCTGATGCGATGCGGTGAAAAGAAGGTAACACTTAACGGAGCCACTACGACGTACCGAGAGTAGATTGGTGAGGAACAGTGGAACGCTCTCATGGTCACAGCACAACGTTCTCAGTTTGAGACTGACGCGAAGCTGAACGAGCAGTATCGCTTGGAGATTAACTCTGCGCTGAACCAAGAGGACCCAAGGACAGCTTGGGAGATGCTTCAAGGTATCAAGGCTGAACTAGATa                                                                                                                                                                                                                          <  1:339010/298‑1 (MQ=255)`  
                                                                                `aaGCCCCACAGCTTAGAAGCCAATCGTTCAGGGACGCTTCTAGCCGTGCTGGTGGTGCTGACGTCCTGATGCGAGTCGGTGACAAGAAGGTAACACTTAACGGAGCCACTACGACTTACCGAGAGTTGATTGGTGAGGAACAGTGGAACGCGCTCATGGTCACAGCACAACGTTCTCAGTTTGAGACTGACGCGAAGCTGAACGAGCAGTATCGCTTGGAGATTAACTCTGCGCTGAACCAAGAGGACCCAAGGACAGCTTGGGAGATGCTTCAAGGTATCAAGGCTGAACTAGATAAGGt                                                                                                                                                                                                                      <  1:180087/301‑1 (MQ=255)`  
                                                                                `aaGCCACCCAGCTTATAAGCCAAGCGTTCAGTGACGCTTCTAGCCGTGCTGGTGGTGCTGACTTCCTGATGCGAGTCGGTGACAAGAAGGTAACACTTAACGGAGCCACTACGACTTACCGAGAGTTGATTGGTGAGGAACAGTGGAACGCTCTCATGGTCACAGCACAACGTTCTCAGTTTGAGACTGACGCGAAGCTGAACGAGCAGTATCGCTTGGAGATTAACTCTGCGCTGAACCAAGAGGACCCAAGGACAGCTTGGGAGATGCTTCAAGGTATCAAGGCTGAACTAGATAAGGt                                                                                                                                                                                                                      <  1:154916/301‑1 (MQ=255)`  
                                                                                `aaGCCACACAGCTTATAAGCCAAGCGTTCAGTGACGCTTCTAGCCGTGCTGGTGGTGCTGACTTCCTGATGCGAGTCGGTGACATGAAGGTAACACTTAACGGAGCCACTACGACTTACCGTTAGTTGATTGGTGAGGAACAGTGGAACGCTCTCATGGTCACAGCACAACGTTCTCAGTTTGAGACTGACGCGAAGCTGAACGAGCAGTATCGCTTGGAGATTAACTCGGCGCTGAACCAAGCGGACCCAAGGACAGCTTGGGAGAGGCTTCCAGGTATCAAGGCTGAACTAGATAAGGt                                                                                                                                                                                                                      >  1:229531/1‑301 (MQ=255)`  
                                                                                `aaGCCACACAGCTTATAAGCCAAGCGTTCAGTGACGCTTCTAGCCGTGCTGGTGGGGCTGACTTCCTGATGCGAGTCGGTGACAAGAAGGTAACACTTAACGGAGCCACTACGACTTACCGAGAGTTGATTGGTGAGGAACAGTGGAACGCTCTCATGGTCACAGCACAACGTTCTCAGTTTGAGACTGACGCGAAGCTGAACGAGCAGTATCGCTTGGAGATTAACTCTGCGCTGAACCAAGAGGACCCAAGGACAGCTTGGGAGATGCTTCAAGGTATCAAGGCTGAACTAGATAAGGt                                                                                                                                                                                                                      <  1:126484/301‑1 (MQ=255)`  
                                                                                     `acacaGCTTATAAGCCAAGCGTTCAGTGACGCTTCTAGCCGTGCTGGTGGTGCTGACTTCCTGATGCGAGTCGGTGACAAGAAGGTAACACTTAACGGAGCCACTACGACTTACCGAGAGTTGATTGGTGAGGAACAGTGGAACGCTCTCATGGTCACAGCACAACGTTCTCAGTTTGAGACTGACGCGAAGCTGAACGAGCAGTATCGCTTGGAGATTAACTCTGCGCTGAACCAAGAGGACCCAAGGACAGCTTGGGAGATGCTTCAAGGTATCAAGGCTGAACTAGAtctgtctcttt                                                                                                                                                                                                                 >  1:206881/1‑291 (MQ=255)`  
                                                                                      `cacaGCTTATAAGCCAAGCGTTCATGGACGCTTCGAGCCGTTCTGGTGGTGCTGACTTCCTGATGCGAGTCGGTGACAAGAAGGTAACACTTAACGGAGCCACTACGACTTACCGAGAGTTGATTGGTGAGTAACAGTGGAACGCTCTCATGGTCACAGCACAACGTTCTCAGTTTGAGACTGACGCGAAGCTGAACGATCAGTATCGCTTGGAGATTAACTCTGCGCTGAACCAAGAGGACCCAAGGACAGCTTGGGAGATGCTTCAAGGTATCAAGGCTGAACTAGATAAGGTCCAAcc                                                                                                                                                                                                                <  1:36881/301‑1 (MQ=255)`  
                                                                                          `gCTTATAAGCCAAGCGTTCAGTGACGCTTCTAGCCGTGCTGGTGGTGCTGACTTCCTGATGCGAGTCGGTGACAAGAAGGTAACACTTAACGGAGCCACTACGACTTACCGAGAGTTGATTGGTGAGGAACAGTGGAGCGCTCTCATGGTCACAGCACAACGTTCTCAGTTTGAGACTGACGCGAAGCTGAACGAGCAGTATCGCTTGGAGATTAACTCTGCGCTGAACCAAGAGGACCCAAGGACAGCTTGGGAGATGCTTCAAGGTATCAAGGCTGAACTAGATAAGGTCCAACCtgat                                                                                                                                                                                                            >  1:146732/1‑301 (MQ=255)`  
                                                                                          `gCTTATAAGCCAAGCGTTCAGTGACGCTTCTAGCCGTGCTGGTGGTGCTGACTTCCTGATGCGAGTCGGTGACAAGAAGGTAACACTTAACGGAGCAACTACGACGTACCGAGAGTTGATGGGTGAGGAACAGTGGAACGCTCTCATGGTCACAGCACAACGTTCTCAGTTTGAGACGGACGCGAAGCTGAACGGGCAGTATCGCTTGGAGATTAAGTCTGCGCTGAACCAAGAGGACCCAAGGACAGCTTGGGAGATGCTTCAAGGTATCAAGGATGAACTAGATAAGGTCCAACCtgat                                                                                                                                                                                                            >  1:115078/1‑301 (MQ=255)`  
                                                                                           `cTTATAAGCCAAGCGTTCAGTGACGCTTCTAGCCGTGCTGGTGGTGCTGACTTCCTGATGCGAGTCGGTGACAAGAAGGTAACACTTAACGGAGCCACTACGACTTACCGAGAGTTGATTGGTGAGGAACAGTGGAACGCTCTCATGGTCACAGCACAACGTTCTCAGTTTGAGACTGACGCGAAGCTGAACGAGCAGTATCGCTTGGAGATTAACTCTGCGCTGAACCAAGAGGACCCAAGGACAGCTTGGGAGATGCTTCAAGGTATCAAGGCTGAACTAGATAAGGTCCAACCtgatg                                                                                                                                                                                                           <  1:75625/301‑1 (MQ=255)`  
                                                                                           `cTTATAAGCCAAGCGTTCAGTGACGCTTCTAGCCGTGCTGGTGGTGCTGACTTCCTGATGCGAGTCGGTGACAAGAAGGTAACACTTAACGGAGCCACTACGACTTACCGAGAGTTGATTGGTGAGGAACAGTGGAACGCTCTCATGGTCACAGCACAACGTTCTCAGTTTGAGACTGACGCGAAGCTGAACGAGCAGTATCGCTTGGAGATTAACTCTGCGCTGAACCAAGAGGACCCAAGGACAGCTTGGGAGATGCTTCAAGGTATCAAGGCTGAACTAGATAAGGTCCAACCtgatg                                                                                                                                                                                                           <  1:252289/301‑1 (MQ=255)`  
                                                                                            `ttATAAGCCAAGCGTTCAGTGACGCTTCTAGCCGTGCTGGTGGTGCTGACTTCCTGATGCGAGTCGGTGACAAGAAGGTAACACTTAACGGAGCCACTACGACTTACCGAGAGTTGATTGGTGAGGAACAGTGGAACGCTCTCATGGTCACAGCACAACGTTCTCAGTTTGAGACTGACGCGAAGCTGAACGAGCAGTATCGCTTGGAGATTAACTCTGCGCTGAACCAAGAGGACCCAAGGACAGCTTGGGAGATGCTTCAAGGTATCAAGGCTGAACTAGATAAGGTCCAACCtgatgc                                                                                                                                                                                                          >  1:345185/1‑300 (MQ=255)`  
                                                                                              `ataAGCCAAGCGTTCAGTGACGCTTCTAGCCGTGCTGGTGGTGCTGACTTCCTGATGCGAGTCGGTGACAAGAAGGTAACACTTAACGGAGCCACTACGACTTACCGAGAGTTGATTGGTGAGGAACAGTGGAACGCTCTCATGGTCACAGCACAACGTTCTCAGTTTGAGACTGACGCGAAGCTGAACGAGCAGTATCGCTTGGAGATTAACTCTGCGCTGAACCAAGAGGACCCAAGGACAGCTTGGGAGATGCTTCAAGGTATCAAGGCTGAACTAGATAAGGTCCAACCTGATGAGc                                                                                                                                                                                                        <  1:1291/301‑1 (MQ=255)`  
                                                                                                   `acAAGAGTTCAGTGAAGCTTCTAGCCGGGCTGGTGGTGCAGACTTCATGATGCGAGTCGGGGACAAGAAGGTAAAACTTAACGGAGCCACTACGACTTACCGAGAGTTGATTGGTGAGGAACAGTGGGACGCTCTCATGGTAAAAGCACAACGTAGTGAGTTTGAGAATGACGAGAAGCTGAAGGAGGAGTATCGCGTGGAGATTAACTGTGCGCTGAACCAAGAGGAGACAAGGACAGCTTGGGAGATGCTTCAAGGTATCAAGGCTGAACTAGATAAGGTCCAACCTGATGAGCAGATg                                                                                                                                                                                                   <  1:305649/300‑1 (MQ=255)`  
                                                                                                      `aGCGTTCAGTGACGCTTCTAGCCGTGCTGGTGGTGCTGACTTCCTGATGCGAGTCGGTGACAAGAAGGTAACACTTAACGGAGCCACTACGACTTACCGAGAGTTGATTGGTGAGGAACAGTGGAACGCTCTCATGGTCACAGCAAAACGTTCTCAGTTTGAGACTGACGCGAAGCTGAACGAGCAGTATCGCTTGGAGATTAACTCTGCGCTGAACCAAGAGGACCCAAGGACAGCTTGGGAGATGCTTCAAGGTATCAAGGCTGAACTAGATAAAGTCCAACCTGATGAGCAGATGaaa                                                                                                                                                                                                >  1:342039/1‑299 (MQ=255)`  
                                                                                                       `gCGTTCAGTGACGCTTCTAGCCGTGCTGGTGGTGCTGACTTCCTGATGCGAGTCGGTGACAAGAAGGTAACACTTAACGGAGCCACTACGACTTACCGAGAGTTGATTGGTGAGGAACAGTGGAACGCTCTCATGGTCACAGCACAACGTTCTCAGTTTGAGACTGACGCGAAGCTGAACGAGCAGTATCGCTTGGAGATTAACTCTGCGCTGAACCAAGAGGACCCAAGGACAGCTTGGGAGATGCTTCAAGGTATCAAGGCTGAACTAGATAAGGTCCAACCTGATGAGCAGATGacac                                                                                                                                                                                               >  1:139026/1‑301 (MQ=255)`  
                                                                                                               `aaagaggcagggCCGTGCTGGTGGGGCTGACTTCCTGATGCGAGTCGGTGACAAGAAGGTAACACTTAACGGAGCCACTACGACTTACCGAGAGTTGATTGGTGAGGAACAGTGGAACGCTCTCATGGTCACAGCACAACGTTCTCAGTTTGAGACTGACGCGAAGCTGAACGAGCAGTATCGCTTGGAGATTAACTCTGCGCTGAACCAAGAGGACCCAAGGACAGCTTGGGAGATGCTTCAAGGTATCAAGGCTGAACTAGATAAGGTCCAACCTGATGAGCAGATGACACCACAACGt                                                                                                                                                                                       <  1:211881/290‑1 (MQ=255)`  
                                                                                                                      `tCTAGCCGTGCTGGTGGTGCTGACTTCCTGATGCGAGTCGGTGACAAGAAGGTAACACTTAACGGAGCCACTACGACTTACCGAGAGTTGATTGGTGAGGAACAGTGGAACGCTCTCATGGTCACAGCACAACGTTCTCAGTTTGAGACTGACGCGAAGCTGAACGAGTAGTATCGCTTGGAGATTAACTCTGCGCTGAACCAAGAGGACCCAAGGACAGCTTGGGAGATGCTTCAAGGTATCAAGGCTGAACTAGATAAGGTCCAACCTGATGAGCAGATGACACCACAACGTGAGTGGc                                                                                                                                                                                <  1:133136/301‑1 (MQ=255)`  
                                                                                                                             `tgtataagagacaggACTGCCTGATGCGAGTCGGTGACAAGAAGGTAACACTTAACGGAGCCACTACGACTTACCGAGAGTTGATTGGTGAGGAACAGTGGAACGCTCTCATGGTCACAGCACAACGTTCTCAGTTTGAGACTGACGCGAAGCTGAACGAGCAGTATCGCTTGGAGATTAACTCTGCGCTGAACCAAGAGGACCCAAGGACAGCTTGGGAGATGCTTCAAGGTATCAAGGCTGAACTAGATAAGGTCCAACCTGATGAGCAGATGACACCACAACGTGAGTGGCTAAtctc                                                                                                                                                                         <  1:39640/287‑1 (MQ=255)`  
                                                                                                                                  `ggtggtGCTGACTTCCTGATGGGAGTCGGTGACAAGAAGGTAACACTTAACGGAGCCACTACGACTTACCGAGAGTTGATTGGTGAGGAACAGTGGAACGCTCTCATGGTCACAGCACAACGTTCTCAGTTTGAGACTGACGCGAAGCTGAACGAGCAGTATCGCTTGGAGATTAACTCTGCGCTGAACCAAGAGGACCCAAGGACAGCTTGGGAGATGCTTCAAGGTATCAAGGCTGAACTAGATAAGGTCCAACCTGATGAGCAGATGACACCACAACGTGAGTGGCTAAtcctgtctc                                                                                                                                                                    >  1:203433/1‑294 (MQ=255)`  
                                                                                                                                   `gtggtgCTGACTTCCTGATGCGAGTCGGTGACAAGAAGGTAACACTTAACGGAGCCACTACGACTTACCGAGAGTTGATTGGTGAGGAACAGTGGAACGCTCTCATGGTCACAGCACAACGTTCTCAGTTTGAGACTGACGCGAAGCTGAACGAGCAGTATCGCTTGGAGATTAACTCTGCGCTGAACCAAGAGGACCCAAGGACAGCTTGGGAGATGCTTCAAGGTATCCAGGCTGAACTAGATAAGGTCCAACCTGCTGAGCAGATGACACCACAACGTGAGTGGCTAATCTCCGcaca                                                                                                                                                                   >  1:56571/1‑301 (MQ=255)`  
                                                                                                                                          `cgACTTCCTGATGCGAGTAGGTGACAAGAAGGTAACACTTAACTGAGCCACTACGACTTACCGAGAGTTGATTGGTGAGGAACAGTGGAACGATATCATGGACACAGCACAACGTTCTCAGTTTGAGACTGACGCGAAGCTGAACGAGCAGTATCGATTGGAGATTAACTCTGCGCTGAACCAAGAGGACCCAAGGACAGCTTGGGAGATGCTTCAAGGTATCAAGGCTGAACTAGATAAGGTCCAACCTGATGAGAAGATGACACCACAACGTGAGTGGCTAATCTCCGCACAGGAACaa                                                                                                                                                            <  1:190440/300‑1 (MQ=255)`  
                                                                                                                                             `cTTCCTGATGCGAGTCGGTGACAAGAAGGTAACACTTAACGGAGCCACTACGACTTACCGAGAGTTGATTGGTGAGGAACAGTGGAACGCTCTCATGGTCACAGCACAACGTTCTCAGTTTGAGACTGACGCGAAGCTGAACGAGCAGTATCGCTTGGAGATTAACTCTGCGCTGAACCAAGAGGACCCAAGGACAGCTTGGGAGATGCTTCAAGGTATCAAGGCTGAACTAGATAAGGTCCAACCTGATGAGCAGATGACACCACAACGTGAGTGGCTAATCTCCGCACAGGAACAAGtt                                                                                                                                                         >  1:80360/1‑301 (MQ=255)`  
                                                                                                                                                             `ggTGACAAGAAGGTAACACTTAACGGAGCCACTACGACTTACCGAGAGTTGATTGGTGAGGAACAGTGGAACGCTCTCATGGTCACAGCACAACGTTCTCAGTTTGAGACTGACGCGAAGCTGAACGAGCAGTATCGCTTGGAGATTAACTCTGCGCTGAACCAAGAGGACCCAAGGACAGCTTGGGAGATGCTTCAAGGTATCAAGGCTGAACTAGATAAGGTCCAACCTGATGAGCAGATGACACCACAACGTGAGTGGCTAATCTCCGCACAGGAACAAGTTCAGAATCAGATGAACg                                                                                                                                         <  1:262659/301‑1 (MQ=255)`  
                                                                                                                                                             `ggTGACAAGAAGGTAACACTTAACGGAGCCACTACGACTTACCGAGAGGTGATTGGTGAGGAACAGTGGAACGCTCTCATGGTCACAGCACAACGTTCTCAGTTTGAGACTGACGCGAAGCTGAACGAGCAGTATCGCTTGGAGATTAACTCTGCGCTGAACCAAGAGGACCCAAGGACAGCTTGGGAGATGCTTCAAGGTATCAAGGCTGAACTAGATAAGGTCCAACCTGATGAGCAGATGACACCACAACGTGAGTGGCTAATCTCCGCACAGGAACAAGTTCAGAATCAGATGAACg                                                                                                                                         >  1:249967/1‑301 (MQ=255)`  
                                                                                                                                                                     `gaagGTAACACTTAACGGAGCCACTACGACTTACCGAGAGTTGATTGGTGAGGAACAGTGGAACGCTCTCATGGTCACAGCACAACGTTCTCAGTTTGAGACTGACGCGAAGCTGAACGAGCAGTATCGCTTGGAGATTAACTCTGCGCTGAACCAAGAGGACCCAAGGACAGCTTGGGAGATGCTTCAAGGTATCAAGGCTGAACTAGATAAGGTCCAACCTGATGAGCAGATGACACCACAACGTGAGTGGCTAATCTCCGCACAGGAACAAGTTCAGaactgtctcttatacacatct                                                                                                                                 >  1:311325/1‑282 (MQ=255)`  
                                                                                                                                                                            `acacTTAACGGAGCCACTACGACTTACCGAGAGTTGATTGGTGAGGAACAGTGGAACGCTCTCATGGTCACAGCACAACGTTCTCAGTTTGAGACTGACGCGAAGCTGAACGAGCAGTATCGCTTGGAGATTAACTCTGCGCTGAACCAAGAGGACCCAAGGACAGCTTGGGAGATGCTTCAAGGTATCAAGGCTGAACTAGATAAGGTCCAACCTGATGAGCAGATGACACCACAACGTGAGTGGCTAATCTCCGCCCAGGAACAAGTTCAGCATCAGATGAACGCATGGACGAAAGCTc                                                                                                                          >  1:123260/1‑301 (MQ=255)`  
                                                                                                                                                                            `acacTTAACGGAGCCACTACGACTTACCGAGAGTTGATTGGTGAGGAACAGTGGAACGCTCTCATGGTCACAGCACAACGTTCTCAGTTTGAGACTGACGCGAAGCTGAACGAGCAGTATCGCTTGGAGATTAACTCTGCGCTGAACCAAGAGGACCCAAGGACAGCTTGGGAGATGCTTCAAGGTATCAAGGCTGAACTAGATAAGGTCCAACCTGATGAGCAGATGACACCACAACGTGAGTGGCTAATCTCCGCACAGGAACAAGTTCAGAATCAGATGAACGCATGGACGAAAGCTc                                                                                                                          >  1:214901/1‑301 (MQ=255)`  
                                                                                                                                                                            `acacTTAACGGAGCCACTACGACTTACCGAGAGTTGATTGGTGAGGAACAGTGGAACGCTCTCATGGTCACAGCACAACGTTCTCAGTTTGAGACTGACGCGAAGCTGAACGAGCAGTATCGCTTGGAGATTAACTCTGCGCTGAACCAAGAGGACCCAAGGACAGCTTGGGAGATGCTTCAAGGTATCAAGGCTGAACTAGATAAGGTCCAACCTGATGAGCAGATGACACCACAACGTGAGTGGCTAATCTCCGCACAGGAACAAGTTCAGAATCAGATGAACGCATGGACGAAAGCTc                                                                                                                          >  1:246013/1‑301 (MQ=255)`  
                                                                                                                                                                             `cacTTAACGGAGCCACTACGACTTACCGAGAGTTGATTGGTGAGGAACAGTGGAACGCTCTCATGGTCACAGCACAACGTTCTCAGTTTGAGACTGACGCGAAGCTGAACGAGCAGTATCGCTTGGAGATTAACTCTGCGCTGAACCAAGAGGACCCAAGGACAGCTTGGGAGATGCTTCAAGGTATCAAGACTGAACTAGATAAGGTCCAACCTGATTAGCAGATGACACCACAACGTGAGTGGCTAATCTCCGCACAGGAACAAGTTCAGacctgtctcttatacacatctccgagccc                                                                                                                         >  1:170265/1‑273 (MQ=255)`  
                                                                                                                                                                             `cacTTAACGGAGCCACTACGACTTACCGAGAGTTGATTGGTGAGGAACAGTGGAACGCTCTCATGGTCACAGCACAACGTTCTCAGTTTGAGACTGACGCGAAGCTGAAAGAGCAGTATCGCTTGGAGATTAACTCTGCGCTGAACCAAGAGGACACAAGGACAGCTTGTGAGATGCTTCCAGGTATCACGGCTGAACTAGATAAGGTCCAACCTGATGAGCAGATGACACCACAACGTGAGTGGCTAATCTACGCACAGGAACAAGTTCAGAATCAGATGAACGCATGGACGAACGCTcc                                                                                                                         >  1:150542/1‑300 (MQ=255)`  
                                                                                                                                                                              `acTTAACGGAGCCACTACGACTTACCGAGAGTTGATTGGTGAGGAACAGTGGAACGCTCTCATGGTCACAGCACAACGTTCTCAGTTTGAGACTGACGCGAAGCTGAACGAGCAGTATCGCTTGGAGATTAACTCTGCGCTGAACCAAGAGGACCCAAGGACAGCTTGGTAGATGCTTCAAGGTATCAAGGCTGAACTAGATAAGGTCCAACCTGATGAGCTGATGACACCACAACGTGAGTGGCTAATCTACGCACAGGCACAAGTTCAGAATCAGAAGAACGCAtctgtctcttataca                                                                                                                        >  1:18304/1‑287 (MQ=255)`  
                                                                                                                                                                              `acTTAACGGAGCCACTACGACTTACCGAGAGTTGATTGGTGAGGAACAGTGGAACGCTCTCATGGTCACAGCACAACGTTCTCAGTTTGAGACTGACGCGAAGCTGAACGAGCAGTATCGCTTGGAGATTAACTCTGCGCTGAACCAAGAGGACCCAAGGACAGCTTGGGAGATGCTTCAAGGTATCAAGGCTGAACTAGATAAGGTCCAACCTGATGAGCAGATGACACCACAACGTGAGTGGCTAATCTCCGCACAGGAACAAGTTCAGAATCAGATGAACGCATGGACGAAAGCTCAg                                                                                                                        >  1:295041/1‑301 (MQ=255)`  
                                                                                                                                                                                `ttAACGGAGCCACTACGACTTACCGAGAGTTGATTGGTGAGGAACAGTGGAACGCTCTCATGGTCACAGCACAACGTTCTCAGTTTGAGACTGACGCGAAGCTGAACGAGCAGTATCGCTTGGAGATTAACTCTGCGCTGAACCAAGAGGACCCAAGGACAGCTTGGGAGATGCTTCAAGGTATCAAGGCTGAACTAGATAAGGTCCAACCTGATGAGCAGATGACACCACAACGTGAGTGGCTAATCTCCGCACAGGAACAAGTTCAGAATCAGATGAACGCATGGACGAAAGCTCAGGc                                                                                                                      <  1:328383/301‑1 (MQ=255)`  
                                                                                                                                                                                      `gatccACTACGACTTACCGAGAGTTGATTGGTGAGGAACAGTGGAACGCTCTCATGGTCACAGCACAACGTTCTCAGTTTGAGACTGACGCGAAGCTGAACGAGCAGTATCGCTTGGAGATTAACTCTGCGCTGAACCAAGAGGACCCAAGGACAGCTTGGGAGATGCTTCAAGGTATCAAGGCTGAACTAGATAAGGTCCAACCTGATGAGCAGATGACACCACAACGTGAGTGGCTAATCTCCGCACAGGAACAAGTTCAGAATCAGATGAACGCATGGACGAAAGCTCAGGCCAAGGc                                                                                                                <  1:160044/298‑1 (MQ=255)`  
                                                                                                                                                                                        `gCCACTACGACTTACCGAGAGTTGATTGGTGAGGAACAGTGGAACGCTCTCATGGTCACAGCACAACGTTCTCAGTTTGAGACTGACGCGAAGCTGAACGAGCAGTATCGCTTGGAGATTAACTCTGCGCTGAACCAAGAGGACCCAAGGACAGCTTGGGAGATGCTTCAAGGTATCAAGGCTGAACTAGATAAGGTCCAACCTGATGAGCAGATGACACCACAACGTGAGTGGCTAATCTCCGCACAGGAACAAGTTCAGAATCAGATGAACGCATGGACGAAAGCTCAGGccctgtctc                                                                                                              >  1:119673/1‑294 (MQ=255)`  
                                                                                                                                                                                        `gCCACTACGACTTACCGAGAGTTGATTGGTGAGGAACAGTGGAACGCTCTCATGGTCACAGCACAACGTTCTCAGTTTGAGACTGACGCGAAGCTGAACGAGCAGTATCGCTTGGAGATTAACTCTGCGCTGAACCAAGAGGACCCAAGGACAGCTTGGGAGATGCTTCAAGGTATCAAGGCTGAACTAGATAAGGCCCAACCTGATGAGCAGATGACACCACAACGTGAGTGGCTAATCTCCGCACAGTAACAAGTTCAGAATCAGATGAACGCATGGACGAAAGCTCAGGCCAAGGctc                                                                                                              >  1:236776/1‑301 (MQ=255)`  
                                                                                                                                                                                                  `atgtgtataagagacagggTGAGGAACAGTGGAACGCTCTCATGGTCACAGCACAACGTTCTCAGTTTGAGACTGACGCGAAGCTGAACGAGCAGTATCGCTTGGAGATTAACTCTGCGCTGAACCAAGAGGACCCAAGGACAGCTTGGGAGATGCTTCAAGGTATCAAGGCTGAACTAGATAAGGTCCAACCTGATGAGCAGATGACACCACAACGTGAGTGGCTAATCTCCGCACAGGAACAAGTTCAGAATCAGATGAACGCATGGACGAAAGCTCAGGCCAAGGCTCTGGACGATTc                                                                                                    <  1:239903/284‑1 (MQ=255)`  
                                                                                                                                                                                                       `cGAGAGTTGATTGGTGAGGAACAGTGGAACGCTCTCATGGGAACAGCACAACGTTCTCAGTTTGAGACTGACGCGAAGCTGAACGAGCAGTATCGCTGGGAGATTAACTCTGCGCTGAACCAAGAGGACCCAAGGACAGCTTGGGAGATGCTTCAAGGTATCAAGGCTGAACTAGATAAGGTCCAACCTGATGAGCAGATGACACCACAACGTGAGTGGCTAATCTCCGCACAGGAACAAGTTCAGAATCAGATGAACGCATGGACGAAAGCTCATGCCAAGGCTCTGGACGATTCCATGa                                                                                               <  1:27225/301‑1 (MQ=255)`  
                                                                                                                                                                                                         `agagTTGATTGGTGAGGAACAGTTGATCGCTCTCATGGTCACAGCACAACGTTCTCAGTTTGAGACTGACGCGAAGCTGAACGAGCAGTATCGCTTGGAGATTAACTCTGCGCTGAACCAAGATGACCCAAGGACAGCTTGAGAGATGCTTCAAGGTATCAAGGCTGAACTAGATAAGGTCCAACCTGATGAGCAGATGACACCACAACGTGAGTGGCTAAACTCCGCACAGGAACAAGTTCAGAATCAGATGAACGCATGGACGAAAGCTCAGGCCAAGGCTCTGGACGATTCCATGAAg                                                                                             <  1:103575/301‑1 (MQ=255)`  
                                                                                                                                                                                                           `agTTGATTGGTGAGGAACAGTGGAACGATCTCATGGTCACAGCACAACGTTCTCAGTTTGAGACTGACGCGAAGCTGAACGAGCAGTATCGCTTGGAGATTAACTCTGCGCTGAACCAAGAGGACCCAAGGACAGCTTGGGAGATGCTTCAAGGTATCAAGGCTGAACTAGATAAGGTCCAACCTGATGAGCAGATGACACCACAACGTGAGTGGCTAATCTCCGCACAGGAACAAGTTCAGAATCAGATGAACGCATGGACGAAAGCTCAGGCCAAGGCTCTGGACGATTCCATGAAGta                                                                                           >  1:53598/1‑300 (MQ=255)`  
                                                                                                                                                                                                                  `tGGTGAGGAACAGTGGAACGCTCTCATGGTCACAGCACAACGTTCTCAGTTTGAGACTGACGCGAAGCTGAACGAGCAGTATCGCTTGGAGATTAACTCTGCGATGAACCAAGAGGACCCAAGGACAGCTTGGGAGATGCTTCAAGGTATCAAGGCTGAACTAGATAAGGTCCAACCTGATGAGCAGATGACACCACAACGTGAGTGGCTAATCTCCGCACAGGAACAAGTTCAGAATCAGATGAACGCATGGACGAAAGCTCAGGCCAAGGCTCTGGACGATTCCATGAAGTCAATGAAc                                                                                    <  1:270505/301‑1 (MQ=255)`  
                                                                                                                                                                                                                   `gaagaagagacagggAACGCTCTCATGGTCACAGAAAAACGTTCTCAGTTTGAGAATGACGCGAAGCTGAACGAGCAGTATCGCTTGGAGATTAACGCTGCGCTGAACCAAGAGGACCCAAGGACAGCTTGGGAGATGCTTCAAGGTATCAAGGCTGAACTAGATAAGGTCCAACCTGATGAGCAGATGACACCACAACGTGAGTGGCTAATCTCCGCACAGGAACAAGTTCAGAATCAGATGAACGCATGGACGAAAGCTCAGGCCAAGGCTCTGGACGATTCCATGAAGTCAATGAACa                                                                                   <  1:321439/288‑1 (MQ=255)`  
                                                                                                                                                                                                                    `gTGAGGAACAGTGGAACGCTCTCATGGTCACAGCACAACGTTCTCAGTTTGAGACTGACGCGAAGCTGAACGAGCAGTATCGCTTGGAGATTAACTCTGCGCTGAACCAAGAGGACCCAAGGACAGCTTGGGAGATGCTTCAAGGTATCAAGGCTGAACTAGATAAGGTCCAACCTGATGAGCAGATGACACCACAACGTGAGTGGCTAATCTCCGCACAGGAACAAGTTCAGAATCAGATGAACGCATGGACGAAAGCTCAGGCCAAGGCTCTGGACGATTCCATGAAGTCAATGAACaa                                                                                  >  1:233668/1‑301 (MQ=255)`  
                                                                                                                                                                                                                         `gAACAGTGGAACGCTCTCATGGTCACAGCACAACGTTCTCAGTTTGAGACTGACGCGAAGCTGAACGAGCAGTATCGCTTGGAGATTAACTCTGCGCTGAACCAAGAGGAACCAAGGACAGCTAGGGAGATGCTTCAAGGTATCAAGGCTGAACTCGATAAGGTCCAACCTGATGAGCAGATGACACCTCAACGTGAGTGGATAATCTCCGCACAGGAACAAGTTCAGAATCAGATTAACGCATGGACGAAAGCTCCAGCCAAGTCTCTGGACGATTCCATGAAGTAATTGAACAAACTTg                                                                             >  1:67705/1‑301 (MQ=255)`  
                                                                                                                                                                                                                            `gtgggctcggagaagtggagaaaaaagagaCGTTAGCAGGTGGATACTGACGGGAAGCAGAACGGGCAGTATCGCAGGGAGATTAACTCTGAGCGGAACCAAGAGGAACCAAGGACAGCTTGGGAGATGCTTCAAGATATCAAGGCTGAACTAGATAAGGTCCAACCTGATGAACAGATGACACCACAACGTGAGTGGCTAATCTCCGCACAGGAACAAGTTCAGAATCAGATGAACGCATGGACGAAAGCTCAGGCCAAGGCTCTGGACGATTCCATGAAGTCAATGAACAAACTTGACg                                                                          <  1:43535/272‑1 (MQ=255)`  
                                                                                                                                                                                                                              `gTGGAACGCTCTCATGGTCACAGCACAACGTTCTCAGTTTGAGACTGACGCGAAGCTGAACGAGCAGTATCGCTTGGAGATTAACTCTGCGCTGAACCAAGAGGACCCAAGGACAGCTTGGGAGATGCTTCAAGGTATCAAGGCTGAACTAGATAAGGTCCAACCTGATGAGCAGATGACACCACAACGTGAGTGGCTAATCTCCGCACAGGAACAAGTTCAGAATCAGATGAACGCATGGACGAAAGCTCAGGCCAAGGCTCTGGACGATTCCATGAAGTCAATGAACAAACTTGACGTa                                                                        >  1:70285/1‑301 (MQ=255)`  
                                                                                                                                                                                                                                      `ctctCATGGTCACAGCACAACGTTCTCAGTTTGAGACTGACGCGAAGCTGAACGAGCAGTATCGCTTGGAGATTAACACTGCGCTGAACCAAGAGGACCCAAGGACAGCTTGGGAGATGCTTCAAGGTATCAAGGCTGCACTAGATAAGGTCCAACCTGATGAGCAGATGACACCACAACGTGAGTGGCTAATCTCCGCACAGGAACAAGTTCAGAATCAGCTGAACGCTTGGACGAAAGCTCAGGCCACGGCTCTGGACGATTCCATGAAGTCAATTAACAAACTTGACGTAATCGACaa                                                                >  1:73576/1‑301 (MQ=255)`  
                                                                                                                                                                                                                                        `ctcATGGTCACAGCACAACGTTCTCAGTTTGAGACTGACGCGAAGCTGAACGAGCAGTATCGCTTGGAGATTAACTCTGCGCTGAACCAAGAGGACCCAAGGACAGCTTGGGAGATGCTTCAAGGTATCAAGGCTGAACTAGATAAGGTCCAACCTGATGAGCAGATGACACCACAACGTGAGTGGCTAATCTCCGCACAGGAACAAGTTCAGAATCAGATGAACGCATGGACGAAAGCTCAGGCCAAGGCTCTGGACGATTCCATGAAGTCAATgctgtctcttatacacatctccgagc                                                              >  1:257411/1‑276 (MQ=255)`  
                                                                                                                                                                                                                                          `cATGGTCACAGCACAACGTTCTCAGTTTGAGACTGACGCGAAGCTGAACGAGCAGTATCGCTTGGAGATTAACTCTGCGCTGAAACAAGAGGACCCAAGGACAGCTTGGGAGATGCTTCAAGGTATCAAGGCTGAACTAGATAAGGTCCAACCTGATGAGCAGATGACACCACAACGTGAGTGGCTAATCTCAGCACAGGAACAAGTTCAGAATCAGATGAACGCATGGACGAAAGCTCAGGCCAAGGCTCTGGACGATTCCATGAAGTCAATGAACAAACTTGACGCAATCGACAAGCaa                                                            >  1:156857/1‑301 (MQ=255)`  
                                                                                                                                                                                                                                              `gTCACAGCACAACGTTCTCAGATTGAGACTGACGCGAAGCTGAACGAGCAGTATCGCTTGGAGATTAACTCTGCGCTGAACCAAGAGGACCCAAGGACAGCTTGGGAGATGCTTCAAGGTATCAAGGCTGAACTAGATAAGGTCCAACCTGATGAGCAGATGACACCACAACGTGAGTGGCTAATCTCCGCACAGGAACAAGTTCAGAATCAGATGAACGCATGGACGAAAGCTCAGGCCAAGGCTCTGGACGATTCCATGAAGTCAATGAACAAACTTGACGTAATCGACAAGCAATTcc                                                        >  1:109702/1‑301 (MQ=255)`  
                                                                                                                                                                                                                                                  `caGCACAACGTTCTCAGTTTGAGACTGACGCGAAGCTGAACGAGCAGTATCGCTTGGAGATTAACTCTGCGCTGAACCAAGAGGACCCAAGGACAGCTTGGGAGATGCTTCAAGGTATCAAGGCTGAACTAGATAAGGTCCAACCTGATGAGCAGATGACACCACAACGTGAGTTGCTAATCTCCGCACCGGAACAAGTTCAGAATCAGATGAACGCATGGACGAAAGCTCAGGCCAAGGCTCTGGACGATTCCATGAAGTCAATGAACAAACTTGACGTAATCGACAAGCAATTCCATaa                                                    >  1:277209/1‑301 (MQ=255)`  
                                                                                                                                                                                                                                                    `gCACAACGTTCTCAGTTTGAGACTGACGCGAAGCTGAACGAGCAGTATCGCTTGGAGATTAACTCTGCGCTGAACCAAGAGGACCCAAGGACAGCTTGGGAGATGCTTCAAGGTATCAAGGCTGAACTAGATAAGGTCCAACCTGATGAGCAGATGACACCACAACGTGAGTGGCTAATCTCCGCACAGGAACAAGTTCAGAATCAGATGAACGCATGGACGAAAGCTCAGGCCAAGGCTCTGGACGATTCCATGAAGTCAATGAACAAACTTGACGTAATCGACAAGCAATTCCAGAAGc                                                  >  1:117622/1‑301 (MQ=255)`  
                                                                                                                                                                                                                                                              `ctcAGTTTGAGACTGACGCGAAGCTGAACGAGCAGTATCGCTTGGAGATTAACTCTGCGCTGAACCAAGAGGACCAAAGGACAGCTTGGGAGATGCTTCAAGGTATCAAGGCTGAACTAGATAAGGTCCAACCTGATGAGCAGATGACACCTCAACGTGAGTGGCTAATCTCCGCACAGGAACAAGTTCAGAATCAGATGAACGCATGGACGAAAGCTCAGGCCAAGGCTCTGGACGATTCCATGAAGTCAATGAACAAACTTGACGTAATCGACAAGCAATTCCAGAAGCGAATCAACgg                                        <  1:50982/301‑1 (MQ=255)`  
                                                                                                                                                                                                                                                               `tcAGTTTGAGACTGACGCGAAGCTGAACGAGCAGTATCGCTTGGAGATTAACTCTGCGCTGAACCAAGAGGACCCAAGGACAGCTTGGGAGATGCTTCAAGGTATCAAGGCTGAACTAGATAAGGTCCAACCTGATGAGCAGATGACACCACAACGTGAGTGGCTAATCTCCGCACAGGAACAAGTTCAGAATCAGATGAACGCATGGACGAAAGCTCAGGCCAAGGCTCTGGACGATTCCATGAAGTCAATGAACAAACTTGACGTAATCGACAAGCAATTCCAGAAGCGAATCAACGGt                                       <  1:87645/301‑1 (MQ=255)`  
                                                                                                                                                                                                                                                                `cAGTTTGAGACTGACGCGAAGCTGAACGAGCAGTATCGCTTGGAGATTAACTCTGCGCTGAACCAAGAGGACCCAAGGACAGCTTGGGAGATGCTTCAAGGTATCAAGGCTGAACTAGATAAGGTCCAACCTGATGAGCAGATGACACCACAACGTGAGTGGCTAATCTCCGCACAGGAACAAGTTCAGAATCAGATGAACGCATGGACGAAAGCTCAGGCCAAGGCTCTGGACGATTCCATGAAGTCAATGAACAAACTTGACGTAATCGACAAGCAATTCCAGAAGctgtctcttatac                                      >  1:97518/1‑289 (MQ=255)`  
                                                                                                                                                                                                                                                                       `agaCTGACGCGAAGCTGAACGAGCAGTATCGCGTGGAGATTAACTCTGCGCTGAACCAAGAGGACCCAAGGACAGCTTGGGAGATGCTTAAAGGTATCAAGGCTGAACTAGATAAGGTCCAACCTGATGAGCAGATGACACCACAACGTGAGTGGCTAATCTCCGCACAGGAACAAGTTCAGAATCAGATGAACGCATGGACGAAAGCTAAGGCCAAGGCTCTGGACGATTCCATGAAGTCAATGAACAAACTTGACGTAATCGACAAGCAATTCCAGAAGCGAATCAACGGTGAGTGGGt                               <  1:29223/301‑1 (MQ=255)`  
                                                                                                                                                                                                                                                                          `cTGACGAGAAGCTGAACGAGCAGTATCGCTTGGAGATTAACTCTGCGCTGAACCAAGAGGACCCAAGGACAGCTTGGGAGATGCTTCAAGGTATCAAGGCTGAACTAGATAAGGTCCAACCTGATGAGCAGATGACACCACAACGTGAGTGGCTAATCTCCGCACAGGAACAAGTTCAGAATCAGATGAACGCATGGACGAAAGCTCAGGCCAAGGCTCTGGACGATTCCATGAAGTCAATGAACAAACTTGACGTAATCGACAAGCAATTCCAGAAGCGAATCAACGGTGAGTGGAtctc                            <  1:282895/301‑1 (MQ=255)`  
                                                                                                                                                                                                                                                                             `aCGCGAAGCTGAACGAGCAGTATCGCTTGGAGATTAACTCTGCGCTGAACCAAGAGGACCCAAGGACAGCTTGGGAGATGCTTCAAGGTATCAAGGCTGAACTAGATAAGGTCCAACCTGATGAGCAGATGACACCACAACGTGAGTGGCTAATCTCCGCACAGGAACAAGTTCAGAATCAGATGAACGCATGGACGAAAGCTCAGTCCAAGGCTCTGGACGATTCCATGAAGTCAATGAACAAACTTGACGTAATCGACAAGCAATTCCAGAAGCAAATCAACGGTGAGTGGGTCTCAAc                         >  1:136032/1‑301 (MQ=255)`  
                                                                                                                                                                                                                                                                                `cgAAGCTGAACGAGCAGTATCGCTTGGAGATTAACTCTGCGCTGAACCAAGAGGACCCAAGGACAGCTTGGGAGATGCTTCAAGGTATCAAGGCTGAACTAGATAAGGTCCAACCTGATGAGCAGATGACACCACAACGTGAGTGGCTAATCTCCGCACAGGAACAAGTTCAGAATCAGATGAACGCATGGACGAAAGCTCAGGCCAAGGCTCTGGACGATTCCATGAAGTCAATGAACAAACTTGACGTAATCGACAAGCAATTCCAGAAGCGAAGCAACGGTGAGTGGGTCTCAACGGa                      >  1:177470/1‑301 (MQ=255)`  
                                                                                                                                                                                                                                                                                  `aaGCTGAACGAGCAGTATCGATTGGAGAGTAACGCTGCGCTGAACCAAGAGGACCCAAGGACAGCTTGGGAGATGCTTCAAGGTATCAATGCTGAACTAGATAAGGTCCAACCTGATGAGCAGATGACACCACAACGTGAGTGGCTAATCTCCGCACAGGAACAAGTTCAGAATCAGATGAACGCATGGACGAAAGCTCAGGCCAAGGCTCTGGACGATTCCATGAAGTCAATGAACAAACTTGACGTAATCGACAAGCAATTCCAGAAGCGAATCAACGGTGAGTGGGTCTCAACGGAtt                    <  1:166495/301‑1 (MQ=255)`  
                                                                                                                                                                                                                                                                                    `gCTGAACGAGCAGTATCGCTTGGAGATTAACTCTGCGCTGAACCAAGAGGACCCAAGGACAGCTTGGGAGATGCTTCAAGGTATCAAGGCTGAACTAGATAAGGTCCAACCTGATGAGCAGATGACACCACAACGTGAGTGGCTAATCTCCGCACAGGAACAAGTTCAGAATCAGATGAACGCATGGACGAAAGCTCAGGCCAAGGCTCTGGACGATTCCATGAAGTCAATGAACAAACTTGACGTAATCGACAAGCAATTCCAGAAGCGAATCAACGGTGAGTGGGTCTCAACGGAtttt                  <  1:325915/301‑1 (MQ=255)`  
                                                                                                                                                                                                                                                                                       `gAACGAGCAGTATCGCTTGGAGATTAACTCTGCGCTGAACCAAGAGGACCCAAGGACAGCTTGGGAGATGCTTCAAGGTATCAAGGCTGAACTAGATAAGGTCCAACCTGATGAGCAGATGACACCACAACGTGAGTGGCTAATCTCCGCACAGGAACAAGTTCAGAATCAGATGAACGCATGGACGAAAGCTCAGGCCAAGGCTCTGGACGATTCCATGAAGTCAATGAACAAACTTGACGTAATCGACAAGCAATTCCAGAAGCGAATCAACGGTGAGTGGGTCTCAACGGATTTTAAg               >  1:254155/1‑301 (MQ=255)`  
                                                                                                                                                                                                                                                                                              `cAGTATCGCTTGGAGATTAACTCTGCGCTGAACCACGAGGACCCAAGGACAGCTTGGGAGATTCTTCAAGGTATCAAGGCTGAACTAGATAAGGTCCAACCTGATGATCAGATGACACCACAACGTGAGTGGCTAATCTCCGCACAGGAACAAGTTCAGAATCAGATGAACGCATGGACGAAAGCTCAGGCCAAGGCTCTGGACGATTCCATGAAGTCAATGAACAAACTTGACGTAATCGACACGAAATTCCAGACGCAAAGCCACGCGCAGTGGGTCTAAACGGATTTTAAGTATATGc        >  1:331790/1‑301 (MQ=255)`  
                                                                                                                                                                                                                                                                                              `cAGTATCGCTTGGAGATTAACTCTGCGCTGAACCAAGAGGACCCAAGGACAGCTTGGGAGATGCTTCAAGGTATCAAGGCTGAACTAGATAAGGTCCCACCTGATGAGCAGATGACACCACAACGTGTGTGGCTAATCTCCGCACAGGAACAAGTTCAGAATCCGATGAACGCATGGACGAAAGCTCAGGCCAAGGCTCTGGACGATTCCATGAAGTCAATGAACAAACTTTACGTAAACGACAAGCAATTCCAGAAGCGAATCAACGGTGAGTGGGTCTCAACTGATTTTTAGGATATGc        >  1:260192/1‑301 (MQ=255)`  
                                                                                                                                                                                                                                                                                              `cAGTATCGCTTGGAGATTAACTCTGCGCTGAACCAAGAGGACCCAAGGACAGCTTGGGAGATGCTTCAAGGTATCAAGGCTGAACTAGATAAGGTCCAACCTGATGAGCAGATGACACCACAACGTGAGTGGCTAATCTCCGCACAGGAACAAGTTCAGAATCAGATGAACGCATGGACGAAAGCTCAGGCCAAGGCTCTGGACGATTCCATGAAGTCAATGAACAAACTTGACGTAATCGACAAGCAATTCCAGAAGCGAATCAACGGTGAGTGGGTCTCAACGGATTTTAAGGATATGc        >  1:106667/1‑301 (MQ=255)`  
                                                                                                                                                                                                                                                                                                  `aTCGCTTGGATATTAACTCTGCGCTGAACCAAGAGGACCCAAGGACAGCTTGGGAGATGCTTCAAGGTATCAAGGCTGAACTAGATAAGGTCCAACCTGATGAGCAGATGACACCACAACGTGAGTGGCTAATCTCCGCACAGGAACAAGTTCAGAATCAGATGAACGCATGGACGAAAGCTCAGGCCAAGGCTCTGGACGATTCCATGAAGTCAATGAACAAACTTGACGTAATCGACAAGCAATTCCAGAAGCGAATCAACGGTGAGTGGGTCTCAACGGATTTTAAGGATATGCCAGt    >  1:317838/1‑301 (MQ=255)`  

                                                                                                                                                                                                                                                                                                          `|`                                                                                                                                                                                                                                                                                                        
`GCGTCGTCCAGACTCTGCTGACTTCTTTGAGAAGTATATCGACAACGGTCTGGTTACTGGCGCAATCCCATCTGATGCTCAAGCCACACAGCTTATAAGCCAAGCGTTCAGTGACGCTTCTAGCCGTGCTGGTGGTGCTGACTTCCTGATGCGAGTCGGTGACAAGAAGGTAACACTTAACGGAGCCACTACGACTTACCGAGAGTTGATTGGTGAGGAACAGTGGAACGCTCTCATGGTCACAGCACAACGTTCTCAGTTTGAGACTGACGCGAAGCTGAACGAGCAGTATCGCTTGAAGATTAACTCTGCGCTGAACCAAGAGGACCCAAGGACAGCTTGGGAGATGCTTCAAGGTATCAAGGCTGAACTAGATAAGGTCCAACCTGATGAGCAGATGACACCACAACGTGAGTGGCTAATCTCCGCACAGGAACAAGTTCAGAATCAGATGAACGCATGGACGAAAGCTCAGGCCAAGGCTCTGGACGATTCCATGAAGTCAATGAACAAACTTGACGTAATCGACAAGCAATTCCAGAAGCGAATCAACGGTGAGTGGGTCTCAACGGATTTTAAGGATATGCCAGTCA  >  NC_001604.1/28960‑29552`

  

Alignment Legend

Aligned base mismatch/match (shaded by quality score): `ATCG/ATCG` `< 3 ≤` `ATCG/ATCG` `< 10 ≤` `ATCG/ATCG` `< 22 ≤` `ATCG/ATCG` `< 34 ≤` `ATCG/ATCG` `< 38 ≤` `ATCG/ATCG`

Unaligned base: `atcg`    Masked matching base: `atcg`    Alignment gap: `‑`    Deleted base: `‑`
